# Audit of Pro Re Nata (PRN) Medication Use in Acute Mental Health Wards in Luton and Bedfordshire

**DOI:** 10.1192/bjo.2026.11752

**Published:** 2026-06-30

**Authors:** Keerthi Vijayan, Sushant Purushothaman

**Affiliations:** East London NHS Foundation Trust, Bedfordshire, United Kingdom

## Abstract

**Aims:**

Pro re nata (PRN) medication is often used on mental health wards to manage agitation, restlessness and risky behaviours. It is important that these medicines are prescribed according to the guidelines and reviewed regularly to ensure patient safety. This audit aims to assess patterns of PRN medication use on mental health wards in Luton and Bedfordshire and to determine whether PRN medications are actively reviewed and discontinued when no longer required.

**Methods:**

We retrospectively reviewed the case records of 25 randomly selected in-patients across General Adult, Old Age and CAMHS wards. Data were collected on patient demographics, diagnosis, legal status, PRN prescriptions, utilisation and administration practices.

**Results:**

The audit showed a high prevalence of PRN medication use, with 84% of patients reviewed prescribed PRN medication. Promethazine and lorazepam were the most frequently prescribed PRN medications, either alone or in combination.10 out of 21 patients were prescribed both promethazine and lorazepam.

There was noticeable variation in PRN utilisation with 6 out of 21 patients requiring frequent PRN administration (more than 20 doses since admission). PRN prescribing appeared appropriate to clinical presentation as approximately 84% of patients requiring higher-frequency PRN use had psychotic symptoms or emotionally unstable personality disorder.

PRN administration adhered to best practice guidance with oral medication regularly offered prior to IM administration.

Areas for improvement were identified: 5 out of 21 patients had PRN prescriptions that were no longer clinically indicated but remained active. PRN protocols were not consistently updated following changes in legal status for 2 patients.

**Conclusion:**

- Introduce regular PRN medication reviews during ward rounds and at defined intervals.
- PRN medications that have not been used for a specified period should be reviewed and discontinued where clinically appropriate.
- PRN protocols should be promptly reviewed and updated following any change in a patient’s legal status.
- Patients requiring frequent PRN medication should receive a multidisciplinary review to explore underlying causes and consider alternative management strategies.
- Improve documentation of PRN indications, review dates and clinical rationale to support safe prescribing.

